# Relevance of body composition in phenotyping the obesities

**DOI:** 10.1007/s11154-023-09796-3

**Published:** 2023-03-17

**Authors:** Laura Salmón-Gómez, Victoria Catalán, Gema Frühbeck, Javier Gómez-Ambrosi

**Affiliations:** 1 Metabolic Research Laboratory, Clínica Universidad de Navarra, Irunlarrea 1, Pamplona, 31008 Spain; 2 CIBER Fisiopatología de la Obesidad y Nutrición (CIBEROBN), Instituto de Salud Carlos III, Pamplona, Spain; 3 Obesity and Adipobiology Group, Instituto de Investigación Sanitaria de Navarra (IdiSNA) Pamplona, Pamplona, Spain; 4 Department of Endocrinology & Nutrition, Clínica Universidad de Navarra, Pamplona, Spain

**Keywords:** Obesity, Body composition, Body fat percentage, Phenotyping, BMI, Waist circumference, Visceral adipose tissue, Metabolic health, Cardiometabolic risk

## Abstract

Obesity is the most extended metabolic alteration worldwide increasing the risk for the development of cardiometabolic alterations such as type 2 diabetes, hypertension, and dyslipidemia. Body mass index (BMI) remains the most frequently used tool for classifying patients with obesity, but it does not accurately reflect body adiposity. In this document we review classical and new classification systems for phenotyping the obesities. Greater accuracy of and accessibility to body composition techniques at the same time as increased knowledge and use of cardiometabolic risk factors is leading to a more refined phenotyping of patients with obesity. It is time to incorporate these advances into routine clinical practice to better diagnose overweight and obesity, and to optimize the treatment of patients living with obesity.

## Introduction

Many of the health advancements gained over the past few decades are now at risk due to the unstoppable increase in the prevalence of obesity that has confirmed to be one of the top causes of disease and death in this century [1]. Despite the alarm raised, the pandemic is expanding unabatedly. In 2015, a total of 108 million children and 604 million adults had obesity. Since 1980, the prevalence of obesity has doubled in more than 70 countries and has continuously increased in most other countries [2]. Overnutrition and a marked decline in physical activity, together with an expanding array of emerging obesogenic factors, are the main causes for this increment [3]. Obesity is a major public health challenge, since it constitutes a risk factor for morbidity and mortality due to its association with pathologies such as type 2 diabetes (T2D), cardiovascular disease (CVD), non-alcoholic fatty liver disease (NAFLD), dyslipidemia, stroke, osteoarticular disorders, obstructive sleep apnea syndrome, and the development of certain types of cancer [4, 5]. It has been suggested that the economic burden of obesity represents between 8.2% and 8.7% of the total health expenses in Western countries [6]. In 2015, high body mass index (BMI) accounted for 4.0 million deaths globally, nearly 40% of which occurred in persons who were not considered as having obesity. More than two thirds of deaths related to high BMI were due to CVD [2].

In recent decades we have witnessed spectacular progress in understanding the etiology as well as the molecular and cellular phenomena that underlie the pathophysiological changes that lead to the development of obesity. However, it seems that these advances have not been translated into clinical practice and nowadays patients with obesity are generally diagnosed and therapeutically managed in a very similar way, as if they all suffered from the same medical condition and all were to respond to treatment in the same manner [7]. Greater accessibility to body composition techniques together with increased knowledge and use of cardiometabolic risk factors currently at our disposal, should allow better phenotyping of patients living with obesity. In this work we review the existing knowledge in relation to the phenotyping of obesity, with special emphasis on the clinical utility of body composition, which we believe could improve the clinical diagnosis and treatment of the “obesities” [8].

## Current obesity classification

The idea of relating height to the square of weight as an anthropometric indicator was first proposed by Quetelet almost 200 years ago [9]. It was not until 1972 that the concept of *body mass index* was coined by Ancel Keys [10]. There has been a longstanding debate among researchers for decades about whether BMI is an adequate tool for the diagnosis of obesity [11]. It is true that, after almost two centuries since its conceptualization, the BMI remains the simplest, cheapest, and easiest to calculate tool for classifying patients with obesity, taking into account their weight and height. The World Health Organization (WHO) and other important international health organizations define overweight as a BMI≥25.0 kg/m^2^ and obesity as a BMI≥30.0 kg/m^2^ (Table 1) [12], although lower cut-off points have been proposed for Asian populations [13]. BMI is very useful in large epidemiological studies [14] but in spite of its widespread use BMI does not accurately reflect body composition and is simply a proxy for measuring body fatness [12, 15–17]. Some researchers believe that the information that BMI provides is not lower or that it is even superior to the body fat percentage (BF%) or other indicators of adiposity such as the fat mass index (fat mass in kg divided by the height in m squared) or muscle mass phenotyping for the prediction of CVD risk [18, 19]. Noteworthy, obesity is defined as a “complication of too much adipose tissue”, and the amount of this excess dysfunctional adiposity is essentially what causes the majority of the health problems linked with obesity [20]. Therefore, identifying the clinical utility of assessing BF% and its usefulness for obesity phenotyping to calculate the cardiometabolic risk linked with obesity may be of outmost importance.

**Table 1:** Cut-off points for obesity classification according to different criteria

**BMI (kg/m** ^**2**^ )*	
< 18.5	Underweight
18.5 - <25.0	Normal weight
25.0 - <30.0	Overweight
≥ 30.0	Obesity
Waist circumference (cm)*	
**Females**	
≥ 80	Increased risk
≥ 88	High risk
**Males**	
≥ 94	Increased risk
≥ 102	High risk
WHR*	
**Females**	
< 0.85	Non-obesity
≥ 0.85	Obesity
**Males**	
< 1.00	Non-obesity
≥ 1.00	Obesity
**WHtR**	
< 0.5	Normal
≥ 0.5	Increased risk
**Body adiposity (%)**	
**Females**	
< 20	Underweight
20 - <30	Normal weight
30 - <35	Overweight
≥ 35	Obesity
**Males**	
< 10	Underweight
10 - <20	Normal weight
20 - <25	Overweight
≥ 25	Obesity

*Different cut-off points have been proposed for Asian populations [13, 149]

BMI, body mass index; WHR, waist-to-hip ratio; WHtR, waist-to-height ratio

## The need to go beyond BMI

It is clear that obesity research has progressed enormously in the last decades using the BMI. However, it has been evidenced that, although being highly useful for epidemiological studies, the use of BMI for the diagnosis and management of obesity has tremendous limitations for the implementation of personalized nutrition in the context of obesity [11, 21]. Because changes in skeletal muscle and other components of lean body mass create significant variances in total body mass, BMI is not a valid clinical tool for determining an individual’s body adiposity [22]. Furthermore, BMI provides misleading information in different conditions such as childhood and adolescence, ageing, intense physical activity, and weight loss including or not exercise [15].

In the era of precision medicine, if we want to refine the clinical management of patients, we need to go beyond the use of BMI and incorporate new tools that allow better classification and follow-up of people living with obesity. In order to improve the phenotyping of patients with obesity, different approaches can be used. The different obesity phenotyping systems that could be implemented include the incorporation of morbidity and functional limitations, genetics, metabolic health, muscular mass, body adiposity and body fat distribution.

## Functional staging systems for obesity

The usefulness of the BMI in obesity classification may be improved by clinical obesity staging systems that incorporate details on the existence and severity of weight-related health problems. Several of them have been proposed, including the King’s Obesity Staging Criteria [23], which was later modified [24] and the Edmonton Obesity Staging System (EOSS) [25]. Since its introduction in 2009, the EOSS has emerged as one of the obesity staging systems that has been the subject of more intense investigation. For instance, several studies have looked into the use of the EOSS to forecast postoperative problems after bariatric surgery and treatment outcomes after conventional dietary or pharmacological obesity treatment. However, these systems do not discriminate different stages in patients with BMI lower than 35 kg/m^2^ or present great variability in their definition and outcomes [24, 26]. The morphofunctional assessment including body composition, functional tests, muscle ultrasound, and laboratory determinations has reportedly shown to provide useful clinical information about the nutritional status of the patients yielding valuable data to better define characteristic phenotypes [27].

## Genetic phenotyping

Genetic analysis performed using genome-wide association studies (GWAS) have revealed that around a hundred loci account for 2.7% of the variation in BMI, and insinuated that as much as 21% of the variation in BMI can be explained by common genetic variation [28]. Other studies have focused on more refined adiposity-related phenotypes, such as BF%, fat-free mass (FFM) or measures of adipose tissue distribution allowing the global identification of more than 500 genetic loci related with obesity [29], although more recent studies describe about a thousand loci related with BMI only [30]. Alleles conferring individual risk do not act independently but rather the interaction among different alleles or between these alleles and the environment is what results in an augmented risk of developing obesity [22]. Genetic variants related with adiposity traits are in general associated with cardiometabolic markers as revealed by epidemiological studies. In this sense, several genetic loci also discovered by GWAS have been shown to uncouple adiposity from its cardiometabolic complications suggesting that therapeutic manipulation of these genes may represent novel therapeutic tools for the reduction of excess adiposity-associated cardiometabolic risk [30]. Recently, the combination of genomics and phenomics has allowed to systematically establish the associations between more than 900 loci related with BMI and more than 1,200 diseases from phenotype codes [31] defined in previous PheWAS analyses [32]. This approach has confirmed the broad impact of obesity on multiple interconnected chronic and acute diseases and opens the door to the establishment of obesity phenotypes by interconnecting genetic variants of obesity with a well-defined extensive disease comorbidity network [31]. More recently, phenome-wide comparative genetic-driven analyses have allowed distinguishing obesity phenotypes with either diabetogenic or antidiabetogenic proclivities based on differences in adiposity distribution, blood pressure and cholesterol content in high-density lipoproteins (HDL) particles [33]. Genetic studies of larger cohorts of common and rare variations and the development of more refined computational tools will allow the identification of additional genetic variants associated with adiposity likely contributing to a better definition of obesity phenotypes. This will help to explain why not all individuals with obesity develop metabolic alterations and to find possible ways to prevent the development of comorbidities in those who are already living with obesity.

## Obesity phenotyping based on BMI and metabolic health

**Fig. 1:**
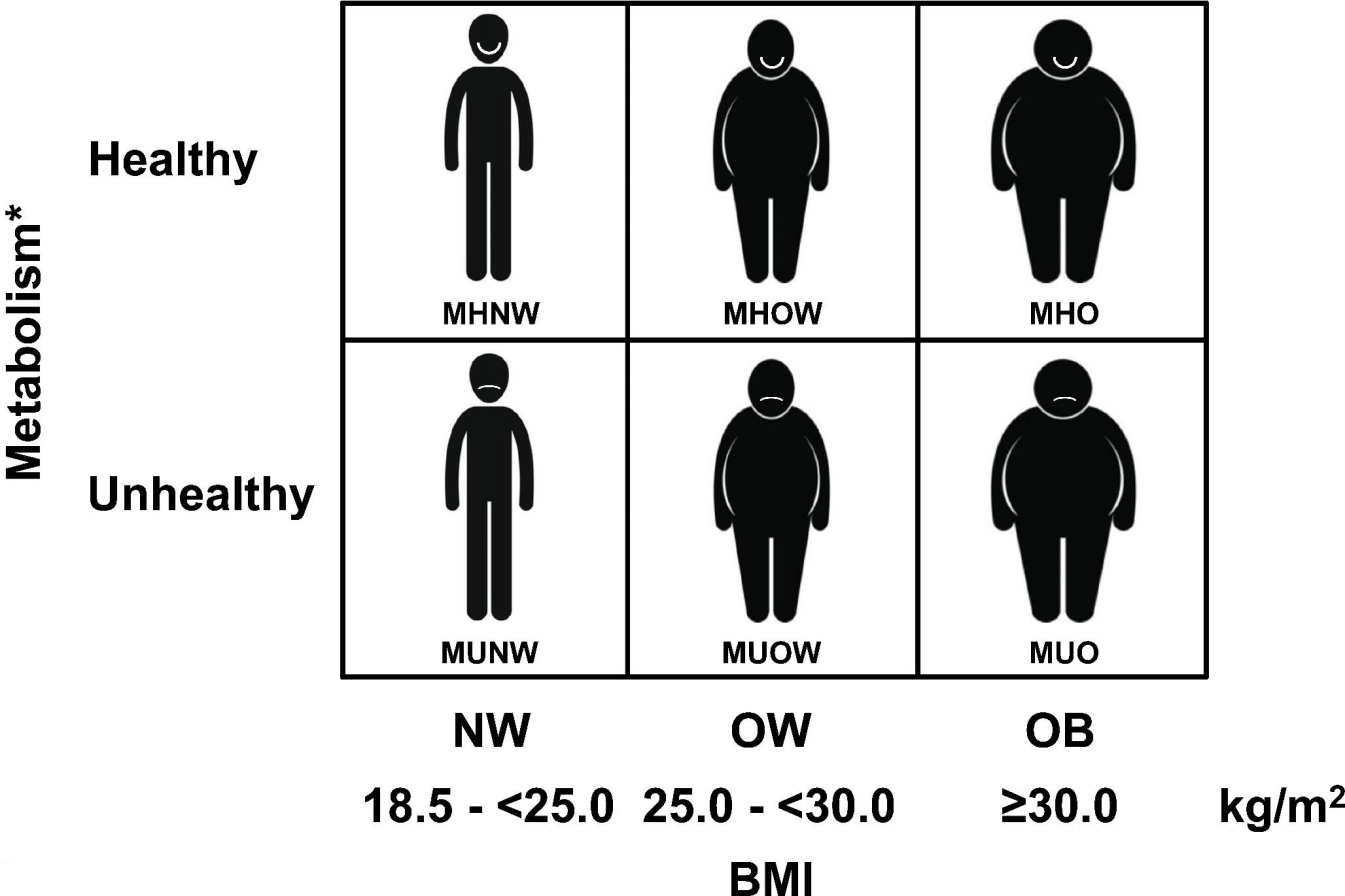
Phenotyping system according to body mass index (BMI) and metabolic health. MHNW, metabolically healthy normal weight (NW); MHOW, metabolically healthy overweight (OW); MHO, metabolically healthy obesity; MUNW, metabolically unhealthy NW; MUOW, metabolically unhealthy OW; MUO, metabolically unhealthy obesity. NW: BMI 18.5 - <25.0 kg/m^2^; OW: BMI 25.0 - <30.0 kg/m^2^; obesity (OB): BMI ≥ 30.0 kg/m^2^. *The criteria for defining healthy vs. unhealthy metabolism are commented in the text

The degree of body adiposity and in particular the accumulation of visceral adipose tissue (VAT) associates with an increased risk of developing obesity-related comorbidities [12, 34, 35]. This is based on the extraordinarily active secretion profile VAT which includes adipokines and factors with a strong cardiometabolic effect [36–41]. However, a proportion of individuals with obesity might not be at increased risk for the development of metabolic alterations and their clinical state has been referred as metabolically healthy obesity (MHO) [34, 42]. In contrast, patients with obesity that at the same time suffer from T2D, hypertension or dyslipidemia are considered as having metabolically unhealthy obesity (MUO or MUHO) [42–44]. Therefore, we may distinguish between healthy and unhealthy individuals with normal weight (NW), overweight or obesity (Fig. 1). Subjects who are NW but metabolically unhealthy (around 20% of the adult population with NW) have a more than 3-fold higher risk of cardiovascular events and/or all-cause death in comparison to those who are NW and metabolically healthy [42, 45]. It is challenging to compare studies regarding MHO/MUO since there is no consensus on its definition, making it almost impossible to estimate the real prevalence of the MHO and MUO phenotypes. In this sense, the reported prevalence of MHO varies widely ranging from 3 to 70% of patients with obesity depending on the method used to define this condition [42, 46–54]. The first studies considered that a patient had MHO if, in addition to having a BMI ≥ 30 kg/m^2^, met less than two conditions related to fasting glycemia, triglycerides, HDL-cholesterol and blood pressure similar to those used to define the metabolic syndrome (MetS) [55]. In the following years there was a very sensible improvement introducing the concept of having none of the MetS components [49, 51]. In our opinion, this is the most appropriate definition since to say that, for example, a patient with obesity and hypertension or with obesity and T2D is healthy is an oxymoron. In addition, transmitting patients with obesity the message that they are healthy can convey a wrong feeling of healthiness conferring them a false sense of security.

Several mechanisms have been proposed to account for the apparently less harmful metabolic profile of subjects with MHO. Among them, a lower inflammatory profile, increased physical activity/higher cardiorespiratory fitness, better renal function, lower uric acid, better sleep pattern, good nutritional status, higher concentrations of adiponectin, reduced adipocyte size or adipose tissue fibrosis and inflammation, or reduced liver fat/liver function have been put forward. Those factors might contribute to the observed differences in the metabolic status among patients with MHO and MUO [42, 51, 53, 56, 57]. Adipose tissue amount and distribution are also determinant factors for the MHO phenotype. It has been clearly established that a greater adipose tissue accumulation in the visceral region contributes to the appearance of the MUO phenotype, while, on the contrary, subcutaneous adipose tissue (SAT) would have a certain protective role against the appearance of this detrimental phenotype [57, 58]. In addition, body composition studies have shown that the MHO is associated with a lower BF% besides a lower VAT [59]. The same is observed in NW people, in whom a lower amount of body adiposity is associated with a healthy phenotype [60].

Many studies have questioned the apparently healthy metabolic profile of MHO suggesting that the risk of comorbidity is lower but not absent [61]. A previous work from our group showed that around 30% of patients considered as with MHO exhibited impaired glucose intolerance or even T2D and that circulating proinflammatory factors levels were similar between individuals with MUO as compared to subjects with MHO, reinforcing the idea that the clinical concept of MHO should be used with caution [62]. Accordingly, adults with MHO show a consistently increased risk of T2D compared to metabolically healthy normal weight (MHNW) subjects across different study populations [63]. In addition, MHO is associated with increased risk of coronary artery calcification [64] and CVD as compared to MHNW [50, 65], even when metabolic health is sustained over a lengthy period of time [66]. Furthermore, MHO has recently shown to confer a higher relative risk for any obesity-related cancer, such as endometrial, liver, renal, and gallbladder cancer, albeit weaker compared to MUO [67].

Some authors consider that the appearance of obesity-associated comorbidities is only a question of temporal evolution of the disease as evidenced by studies showing that subjects defined as with MHO show higher risk of developing T2D, atherosclerosis, hypertension or MetS in the long-term [42, 68–70]. In this sense, a growing number of publications have questioned the seemingly healthy metabolic condition of MHO evidencing that these patients with obesity exhibit increased morbidity and mortality as compared to MHNW [63, 65, 71–74].

MHO, therefore, should not be viewed as an obesity that is safe and does not require treatment, but it may help in guiding decision-making for a customized and risk-based treatment of obesity [42]. Since the distinction between the various obesity phenotypes may have significant therapeutic implications, a proper definition for the stratification of people living with obesity and an accurate diagnosis are crucial for the individualized care of these patients. A better definition of the obesity subphenotypes and a precise diagnosis that more accurately identifies the actual metabolic state together with the function and expansion capacity of adipose tissue without incurring into the contradiction of applying the term healthy when actually metabolic derangements are already present both at circulating and tissue level are needed to improve the management of patients with obesity.

## Phenotyping of obesity based on anthropometric measurements different to BMI

Although the BMI has been shown to be a powerful tool to classify patients according to their adiposity, with the limitations that have been commented above, and to somehow estimate their cardiometabolic risk, it seems clear that the distribution of adiposity should also be taken into account in the management of patients with obesity [75–78]. In this sense, more than seven decades have passed since the introduction in the forties of the notion that VAT has a much greater pathogenic effect than SAT, that may even exert a certain protective effect [79]. In this sense, simple to obtain measurements as waist circumference (WC) have shown to relatively estimate the amount of VAT being good indicators of both morbidity and mortality [80–82].

**Fig. 2:**
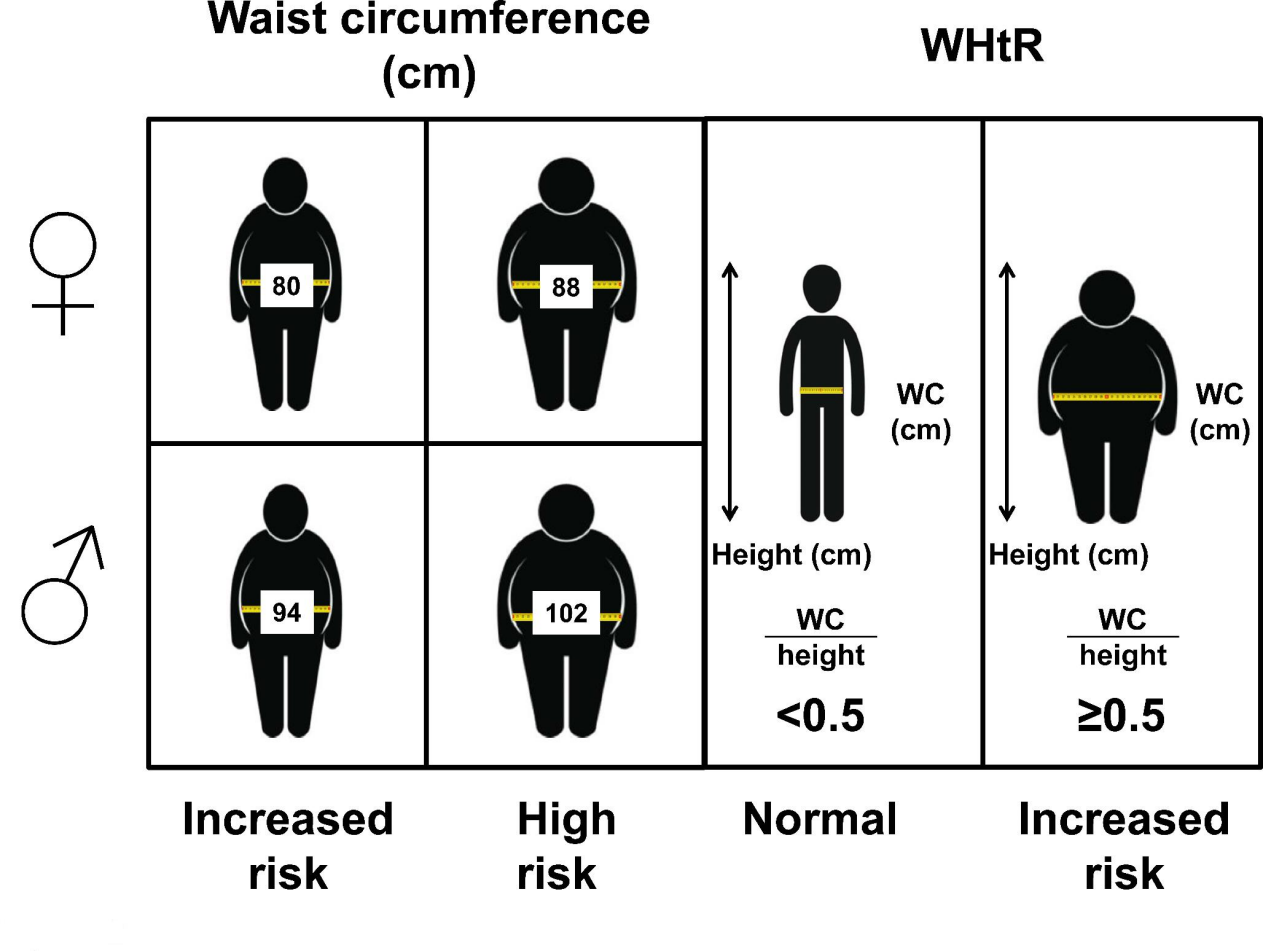
Threshold values to estimate cardiometabolic risk according to waist circumference for females and males (left) and waist-to-height ratio (WHtR, right)

Although there is no clear consensus on the anatomical point where to correctly measure the WC, the protocol used does not seem to have a substantial influence on the estimation of the associated cardiometabolic risk [83]. Global cut-off points to define increased or high cardiometabolic risk have been established (Table 1; Fig. 2), although optimized values for specific ethnicities have been proposed later [83]. WC thresholds within each BMI category for the estimation of CVD risk have been also proposed [84]. Several studies have analyzed the impact on the obesity-associated cardiometabolic risk by stratifying patients according to both BMI and WC, showing that taking into account estimators of body adiposity and distribution produces a marked improvement in the predictive capacity over those measures considered separately [80, 81, 85–88]. However, well defined and consensus phenotypes according to the combined use of BMI and WC have not been established so far.

Another frequently used anthropometric estimator of abdominal adiposity is the waist-to-hip ratio (WHR), which is calculated dividing the WC by the hip circumference both in cm. According to the WHO, the WHR cut-off points to detect obesity are ≥ 0.85 and ≥ 1.0 in females and males, respectively (Table 1). WHR has been shown to have a similar capacity than BMI or WC in predicting incident T2D in prospective studies [89, 90], although WC and WHR seems to discriminate better than BMI the presence of T2D in cross-sectional studies [90]. Several meta-analyses reported that WC and WHR similarly predict cardiovascular events and mortality better than the BMI [91, 92], although more recent studies suggest than WC is a better predictor of heart failure than WHR [93]. Moreover, WC has been shown to be a better estimator of VAT than the WHR [94]. However, these epidemiological studies analyze more the effect of the WC and the WHR measured as continuous variables than the stratification according to the cut-off points defined to phenotype obesity. Therefore, it is difficult to draw a consistent conclusion about their usefulness with the proposed phenotyping thresholds. Several studies have also used the combined influence of BMI and WHR to predict CVD and mortality risk [80, 87].

In the last years, another estimator of adiposity that has been gaining interest is the waist-to-height ratio (WHtR) calculated dividing the WC by the height both expressed in cm [95]. A boundary value of 0.5 for WHtR has been suggested by Ashwell et al. being now frequently used [96]. This is equivalent to the straightforward screening instruction “keep your waist to less than half your height” (Table 1 and Fig. 2). This message not only seems appropriate for all racial and ethnic groups, but it is also suitable for being used with children [96]. The use of WHtR for the screening of adults at increased cardiometabolic risk has been shown to perform better than BMI by meta-analyses [97] and has been suggested to be a more useful clinical screening tool than WC [98–100] or a combination of BMI and WC [101]. A modification of this index has been proposed: the WC index (WC/height^0.5^) having stronger association with adiposity, but its clinical usefulness needs to be further explored [102].

Despite the fact that the WC is easy to obtain and inexpensive, it is worth mentioning that while being a good indicator of abdominal adiposity its correlation with VAT determined through imaging techniques such as computed tomography (CT) or magnetic resonance imaging (MRI) is low, since it does not discriminate between SAT and VAT, abdominal adipose depots with very different pathophysiological implications. However, these imaging techniques are expensive, require a well-trained observer, and are not always available in the clinical practice [77].

## Body composition and obesity phenotyping

**Fig. 3:**
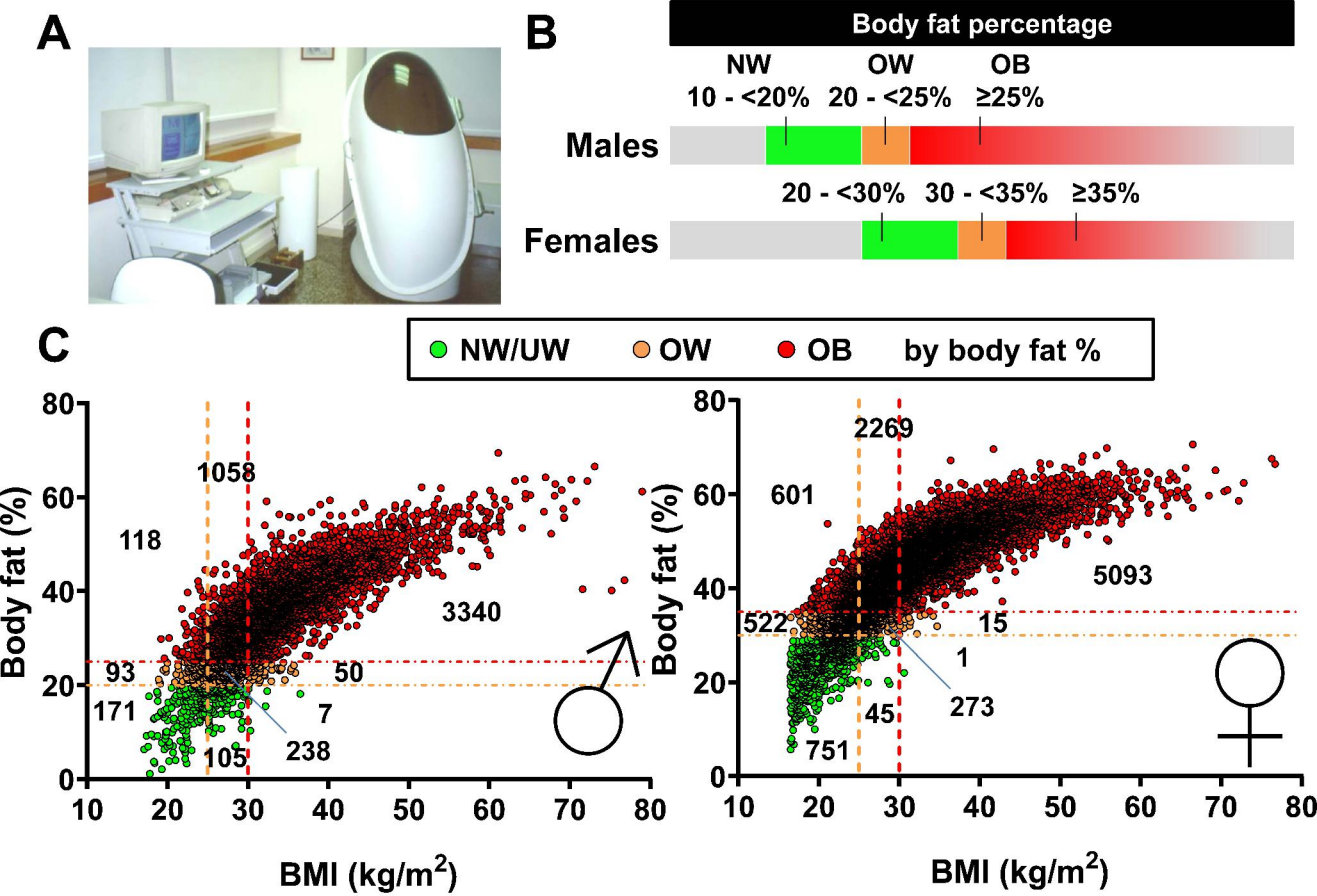
Body mass index (BMI) misclassifies a high number of patients with overweight or obesity defined by body fat percentage (BF%). (A) Air displacement plethysmography equipment used to estimate BF% in people with a BMI ≥ 16.5 kg/m^2^ attending the Department of Endocrinology and Nutrition at the Clínica Universidad de Navarra in Pamplona, Spain. (B) Cut-off points used to define overweight and obesity according to BF% in men and women. (C) Correlation between BMI and BF% of a sample of 14,750 individuals stratified by gender. Left: Men (n = 5,180). Right: Women (n = 9,570). Vertical dashed lines indicate cut-offs for defining overweight (OW) and obesity (OB) according to BMI (25.0 and 30.0 kg/m^2^, respectively) while horizontal lines indicate cut-offs for defining OW and OB according to BF% (20.0 and 25.0% in males and 30.0 and 35.0% in females, respectively). The number of subjects in each quadrant is indicated. Colors denote normal weight/underweight (NW/UW), OW or OB according to BF%

Obesity is defined as an excess of adiposity, with the amount of this excess correlating with comorbidity development [8]. Although the BMI shows a good correlation with adiposity in large population studies, it presents a very high error rate when we study patients at the individual level; this fact is very remarkable in the era of personalized medicine. In this sense, we found that almost a third of subjects classified as having NW according to BMI and around 80% of subjects considered as with overweight according to BMI exhibited a BF% that would make them to be considered as having obesity, as can be observed in Fig. 3 with data obtained using air displacement plethysmography. Moreover, these incorrectly classified patients exhibited numerous risk factors above the thresholds established for predicting cardiometabolic risk. However, only a few of the subjects with a BF% within the NW or overweight range was misclassified as having obesity according to the BMI value [12]. Our data, together with other studies [16, 17] evidence that there is a substantial degree of misclassification in the diagnosis of obesity in clinical practice using the BMI, in particular in those considered as having overweight, and that we are missing opportunities to treat patients with this life-threatening condition.

The study of body composition for determining BF% can be approached from very varied techniques including skin-fold measurement, bioelectrical impedance analysis (BIA), dual-energy X-ray absorptiometry, air displacement plethysmography, MRI, CT, isotopic dilution or underwater weighing [103–105]. The availability of devices to determine body composition has been increasing in recent years and nowadays it is very common to have them (for example BIA devices, whose accuracy has increased over the years) available in consultations with nutritionists, endocrinologists or even in primary care offices.

Excess adiposity measured as BF% correlates very well with the increase in the risk of CVD, T2D and other obesity-associated comorbidities [106–109]. Most of the studies aimed to determine the influence of adiposity on cardiometabolic alterations have focused more on estimators of body fat distribution than on the amount of body fat per se. However, a growing number of studies indicate that the amount of body fat is also exerting a fundamental role in the increased cardiometabolic risk [12, 36, 110–115]. Body composition provides a scientific explanation that may help to understand the observed increased cardiovascular risk in metabolically unhealthy normal weight (MUNW) subjects with high adiposity [43, 116, 117]. In this sense, the use of BF% thresholds for the diagnosis of obesity (Table 1) allows to detect more subjects with an increased cardiometabolic risk than the simple application of the BMI or the WC classification criteria. Those cut-off points for BF% used for females (30 - <35% defining overweight and ≥ 35 defining obesity) and males (20 - <25% defining overweight and ≥ 25 defining obesity) are frequently used in the literature [12, 16, 17, 112, 118–122], even in children and adolescents [123–125], although an international consensus does not exist.

This is of particular relevance due to the pathophysiological implications that increased adiposity may have in the context of NW or overweight. Although BMI is widely used as a proxy indicator of body adiposity, it does not provide an actual measure of body composition as previously evidenced [12]. A high number of patients with obesity are being underdiagnosed, and, therefore, opportunities for cardiometabolic risk assessment and instauration of appropriate treatment measures are being lost. In this sense, the inclusion of body composition determination together with the evaluation of metabolic alterations in the routine clinical practice both for the diagnosis and the instauration of the most adequate management of obesity should be pursued [8].

## Phenotyping visceral obesity with imagen techniques

An elevated VAT is a hallmark sign of increased cardiometabolic risk, even among NW subjects [126–128]. Imaging techniques using first CT and MRI thereafter revealed that the amount of VAT is a major determinant of the cardiometabolic risk [129, 130]. There is no consensus for VAT area cut-off points to define increased metabolic risk, although it has been proposed that in both females and males a value of 100 cm^2^ was linked to significant changes in the risk profile for CVD, and when values of more than 130 cm^2^ of VAT were attained, a further elevation of the cardiometabolic risk was seen [131]. In addition, these techniques have been improved and volumetric data obtained from multislice imaging has confirmed that even after taking into consideration the usual anthropometric measures VAT is still more strongly linked to a harmful cardiometabolic risk profile [132]. Therefore, VAT determination may provide a more detailed picture of the obesity-associated cardiometabolic risk. Furthermore, imaging techniques allowed the identification of a new subphenotype of subjects with NW corresponding to individuals with a BMI < 25 kg/m^2^ with an intra-abdominal adipose tissue/abdominal subcutaneous adipose tissue ratio above 0.45 in women and 1.0 in men, that would have and increased cardiometabolic risk [133]. This phenotype was named TOFI (standing for “thin-on-the-outside fat-on-the-inside”) representing people with NW but an abnormally high amount of “hidden” VAT [133], that could contribute to explain the MUNW phenotype.

## Skeletal muscle mass and obesity: sarcopenic obesity

**Fig. 4:**
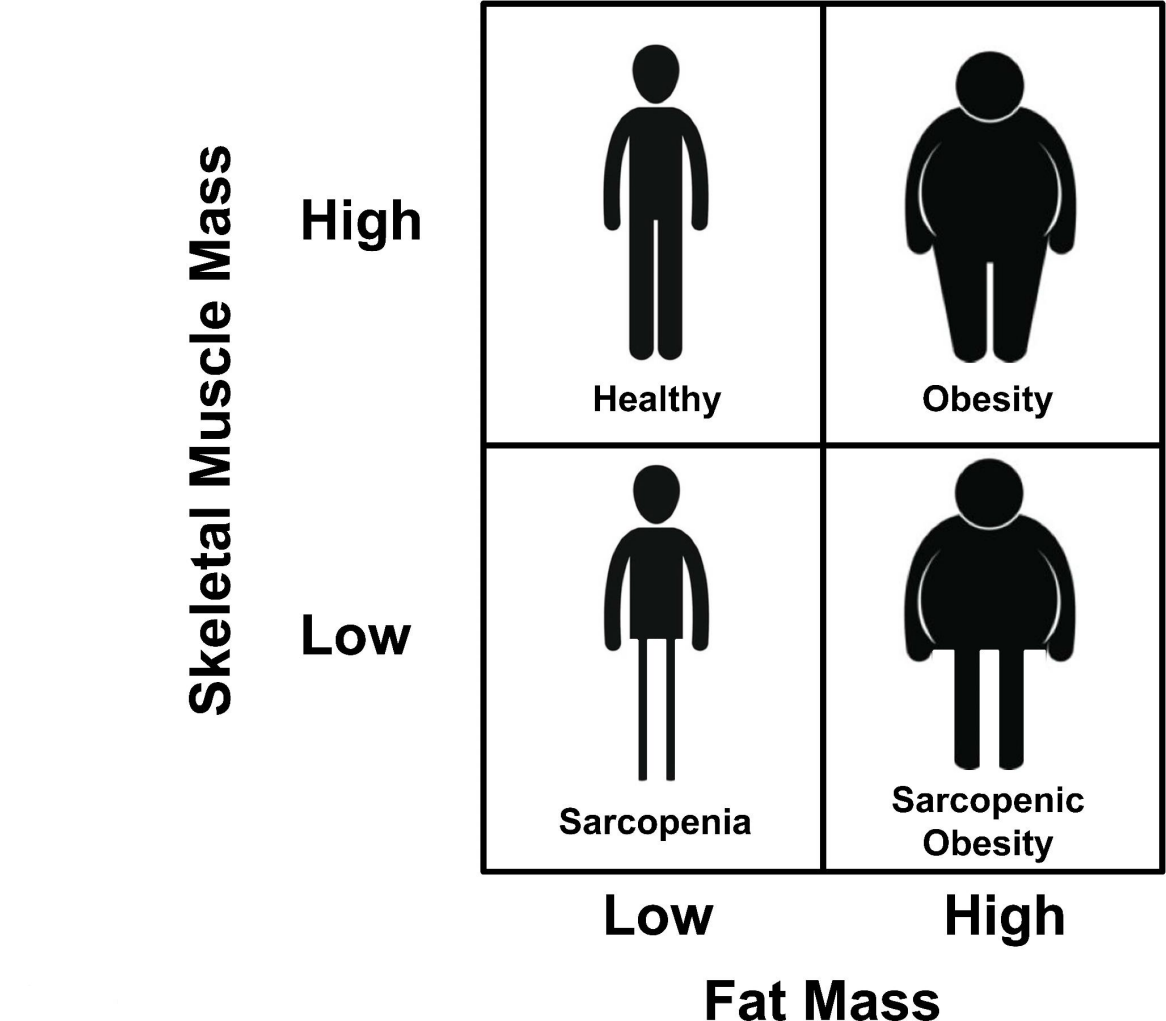
Phenotyping system according to fat mass and skeletal muscle mass. The evaluation regarding skeletal muscle mass includes amount and functionality. The diagnostic criteria are reported in a consensus statement [136]

The information obtained thanks to body composition techniques has allowed to detect the presence of a condition with important functional implications that consists of the simultaneous presence of excess adiposity and a deficit in skeletal muscle mass and function (sarcopenia), which has been defined as sarcopenic obesity [134–136]. The matrix resulting from the combination of low and high body adiposity and low and high skeletal muscle mass results in the establishment of another classification system for obesity-related phenotypes (Fig. 4). The diagnosis is based on skeletal muscle functional parameters (for example hand-grip strength adjusted by body mass) and, if a dysfunction is detected, the process will continue with body composition to identify potential increased fat mass and reduced skeletal muscle mass. When both situations concur the presence of sarcopenic obesity can be diagnosed [136]. This medical condition, which has a global prevalence of around 11% in older adults [137], has been attributed to the consequences of the ageing process, acute and chronic diseases, and the lack of physical activity [136], and is associated with an increase in mortality [138]. The lack of clear diagnostic criteria during the last years made it difficult to adequately study the cardiometabolic risk associated with these phenotypes. To solve the lack of clear criteria, “alternative” diagnostic premises have been generated through machine learning. With this approach it was shown that sarcopenic obesity is associated with an increase in cardiometabolic risk [139, 140]. In the same line, other studies using this phenotyping system have suggested that the maintenance of skeletal muscle mass with ageing reduced the development of T2D [141].

## A new phenotyping classification combining body fat amount and distribution

**Fig. 5:**
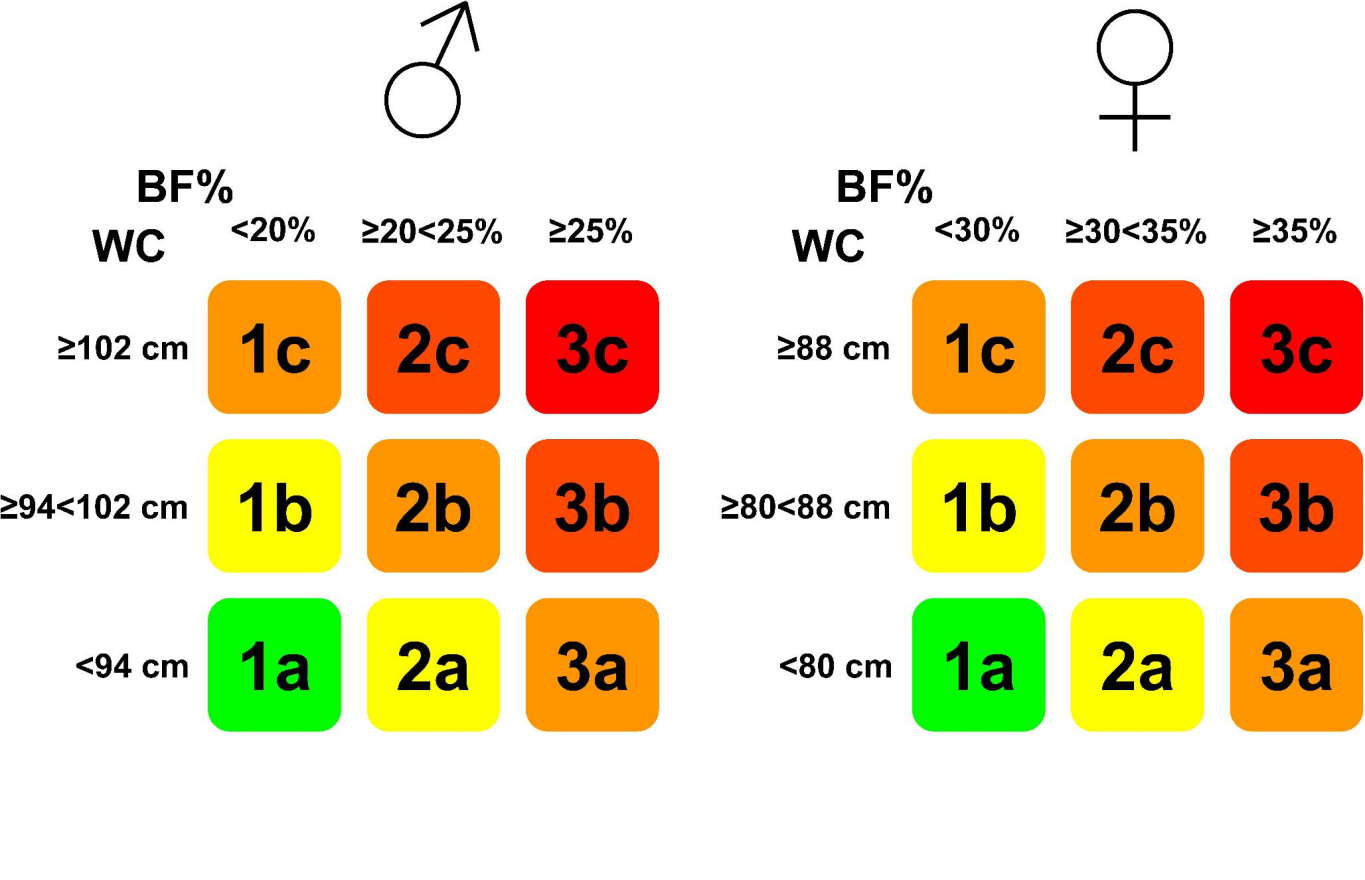
Proposed phenotyping system based on a combination of the actual adiposity expressed as body fat percentage (BF%) and waist circumference (WC) as a measure of adiposity distribution. The cutoff points are those defined by the WHO for WC and the most frequently used for BF% (see text). This phenotyping system establishes nine different types (1a to 3c) clustered in five different phenotypes according to the cardiometabolic risk. Green: no risk; yellow: slightly increased risk; orange: increased risk; dark orange: high risk and red: very high risk

Both BMI and WC may have useful applications in routine clinical practice. However, they bear a high error rate, as previously mentioned, being anthropometric-based classification systems that are only useful for predicting health hazards and do not reveal accurate information about a patient’s health situation or clinical need [26, 103, 104, 142]. As commented above, patient stratification according to BMI and WC simultaneously allows a better prediction of cardiovascular or death risk [80, 81, 85–87]. However those studies were not aimed to establish different phenotypes according to both amount and distribution of adiposity. Other studies have used the combination of BMI and WC to define a kind of “matrix” to establish specific cardiometabolic risk phenotypes [101, 143]. However, although the cardiometabolic risk estimated with this approach is more precise than the use of BMI or WC alone it still maintains the mentioned BMI limitations. We herein propose that a combination of the actual adiposity expressed as BF% and WC as a measure of distribution may represent a novel and useful tool for the estimation of obesity-associated cardiometabolic risk both for research, but also in the clinical setting, getting a more precise insight and providing a better translation into increased risk. This phenotyping system (Fig. 5) establishes nine body phenotypes (3 BF% x 3 WC) grouped in five different risk phenotypes, following a traffic light-like classification system (green: no risk; yellow: slightly increased risk; orange: increased risk; dark orange: high risk and red: very high risk) according to the predicted risk. By using this approach, a very accurate stratification of cardiometabolic risk factors is achieved (unpublished results). Moreover, since information provided by body composition techniques is not always available we suggest to combine BF% directly measured or estimated by body fat equations, such as the CUN-BAE (Clínica Universidad de Navarra-Body Adiposity Estimator), which provides a better appraisal of actual BF% than BMI [144], with the measurement of WC.

## Conclusions

It seems clear that the current obesity classification systems do not allow a good diagnosis and prediction of the comorbidity risk of the patients and, therefore, their clinical management. More than a decade ago it was proposed that obesity phenotyping should be more sophisticated including dynamic rather than static phenotypes taking into account the variability in body composition and its influence on metabolism and the physiological responses to diet and exercise [145]. Some advances have been achieved in the phenotyping of obesities with the incorporation, for example, of functionality or metabolic health. Moreover, the evolution of software tools is allowing the development of new approaches incorporating machine learning for establishing new classification systems according to the combination of genetics, adipocyte morphology and metabolic traits [146], the calculation of adiposity by using smartphones [147] or the development of whole-body three-dimensional optical scanning to predict the metabolic risk [148]. Improvements in the resolution and availability of body composition equipments have also produced a greater implementation of these techniques. Body composition has allowed the identification of sarcopenic obesity, to establish more refined obesity phenotypes and to better define obesity-associated cardiometabolic risk. It is time to implement these advances in routine clinical practice in a more constant way to prevent the development of overweight and obesity, as well as to achieve a better management of people living with obesity.

## Abbreviations

BIA: bioelectrical impedance analysis
BF%: body fat percentage
BMI: body mass index
CT: computed tomography
CUN-BAE: Clínica Universidad de Navarra-Body Adiposity Estimator
CVD: cardiovascular disease
EOSS: Edmonton Obesity Staging System
FFM: fat-free mass
HDL: high-density lipoproteins
GWAS: genome-wide association studies
MetS: metabolic syndrome
MHNW: metabolically healthy normal weight
MHO: metabolically healthy obesity
MHOW: metabolically healthy overweight
MRI: magnetic resonance imaging
MUHO: metabolically unhealthy obesity
MUNW: metabolically unhealthy normal weight
MUO: metabolically unhealthy obesity
MUOW: metabolically unhealthy overweight
NAFLD: non-alcoholic fatty liver disease
NW: normal weight
SAT: subcutaneous adipose tissue
T2D: type 2 diabetes
VAT: visceral adipose tissue
WC: waist circumference
WHR: waist-to-hip ratio
WHtR: waist-to-height ratio
WHO: World Health Organization

## References

[1] Blüher M (2019). Obesity: global epidemiology and pathogenesis. Nat Rev Endocrinol.

[2] The GBD 2015 Obesity Collaborators (2017). Health effects of overweight and obesity in 195 countries over 25 years. N Engl J Med.

[3] Catalán V, Avilés-Olmos I, Rodríguez A, Becerril S, Fernández-Formoso JA (2022). Time to consider the “Exposome Hypothesis” in the development of the obesity pandemic. Nutrients.

[4] Bray GA, Heisel WE, Afshin A, Jensen MD, Dietz WH (2018). The science of obesity management: an Endocrine Society scientific statement. Endocr Rev.

[5] Frühbeck G, Busetto L, Dicker D, Yumuk V, Goossens GH (2019). The ABCD of obesity: an EASO position statement on a diagnostic term with clinical and scientific implications. Obes Facts.

[6] OECD. (2019) The heavy burden of obesity. The economics of prevention. Acceded January 2023. http://www.oecd.org/health/the-heavy-burden-of-obesity-67450d67-en.htm.

[7] Yárnoz-Esquiroz P, Olazarán L, Aguas-Ayesa M, Perdomo CM, García-Goni M (2022). Obesities’: position statement on a complex disease entity with multifaceted drivers. Eur J Clin Invest.

[8] Perdomo CM, Cohen RV, Sumithran P, Clement K, Frühbeck G (2023). Contemporary medical, device, and surgical therapies for obesity in adults. Lancet.

[9] Quetelet LAJ (1835). Sur l’homme et le development de ses facultes, ou essai de physique sociale.

[10] Keys A, Fidanza F, Karvonen MJ, Kimura N, Taylor HL (1972). Indices of relative weight and obesity. J Chronic Dis.

[11] Blundell JE, Dulloo AG, Salvador J, Frühbeck G (2014). Beyond BMI – phenotyping the obesities. Obes Facts.

[12] Gómez-Ambrosi J, Silva C, Galofré JC, Escalada J, Santos S (2012). Body mass index classification misses subjects with increased cardiometabolic risk factors related to elevated adiposity. Int J Obes.

[13] WHO Expert Consultation (2004). Appropriate body-mass index for asian populations and its implications for policy and intervention strategies. Lancet.

[14] Kivimäki M, Strandberg T, Pentti J, Nyberg ST, Frank P (2022). Body-mass index and risk of obesity-related complex multimorbidity: an observational multicohort study. Lancet Diabetes Endocrinol.

[15] Prentice AM, Jebb SA (2001). Beyond body mass index. Obes Rev.

[16] Romero-Corral A, Somers VK, Sierra-Johnson J, Thomas RJ, Collazo-Clavell ML (2008). Accuracy of body mass index in diagnosing obesity in the adult general population. Int J Obes.

[17] Okorodudu DO, Jumean MF, Montori VM, Romero-Corral A, Somers VK (2010). Diagnostic performance of body mass index to identify obesity as defined by body adiposity: a systematic review and meta-analysis. Int J Obes.

[18] Ortega FB, Sui X, Lavie CJ, Blair SN. Body mass index, the most widely used but also widely criticized index: Would a criterion standard measure of total body fat be a better predictor of cardiovascular disease mortality? Mayo Clin Proc. 2016;91:443–455. 10.1016/j.mayocp.2016.01.008 PMC4821662 26948431

[19] Kakinami L, Danieles PK, Ajibade K, Santosa S, Murphy J (2021). Adiposity and muscle mass phenotyping is not superior to BMI in detecting cardiometabolic risk in a cross-sectional study. Obes (Silver Spring).

[20] Cypess AM (2022). Reassessing human adipose tissue. N Engl J Med.

[21] Frühbeck G, Kiortsis DN, Catalán V (2018). Precision medicine: diagnosis and management of obesity. Lancet Diabetes Endocrinol.

[22] Schwartz MW, Seeley RJ, Zeltser LM, Drewnowski A, Ravussin E (2017). Obesity pathogenesis: an endocrine society scientific statement. Endocr Rev.

[23] Aylwin S, Al-Zaman Y (2008). Emerging concepts in the medical and surgical treatment of obesity. Front Horm Res.

[24] Aasheim ET, Aylwin SJ, Radhakrishnan ST, Sood AS, Jovanovic A (2011). Assessment of obesity beyond body mass index to determine benefit of treatment. Clin Obes.

[25] Sharma AM, Kushner RF (2009). A proposed clinical staging system for obesity. Int J Obes.

[26] Atlantis E, Sahebolamri M, Cheema BS, Williams K (2020). Usefulness of the Edmonton obesity staging system for stratifying the presence and severity of weight-related health problems in clinical and community settings: a rapid review of observational studies. Obes Rev.

[27] García Almeida JM, García García C, Vegas Aguilar IM, Bellido Castañeda V, Bellido Guerrero D (2021). Morphofunctional assessment of patient s nutritional status: a global approach. Nutr Hosp.

[28] Locke AE, Kahali B, Berndt SI, Justice AE, Pers TH (2015). Genetic studies of body mass index yield new insights for obesity biology. Nature.

[29] Loos RJ (2018). The genetics of adiposity. Curr Opin Genet Dev.

[30] Huang LO, Rauch A, Mazzaferro E, Preuss M, Carobbio S (2021). Genome-wide discovery of genetic loci that uncouple excess adiposity from its comorbidities. Nat Metab.

[31] Huang J, Huffman JE, Huang Y, Do Valle I, Assimes TL (2022). Genomics and phenomics of body mass index reveals a complex disease network. Nat Commun.

[32] Denny JC, Ritchie MD, Basford MA, Pulley JM, Bastarache L (2010). PheWAS: demonstrating the feasibility of a phenome-wide scan to discover gene-disease associations. Bioinformatics.

[33] Coral DE, Fernandez-Tajes J, Tsereteli N, Pomares-Millan H, Fitipaldi H et al. A phenome-wide comparative analysis of genetic discordance between obesity and type 2 diabetes.Nat Metab. 2023. 10.1038/s42255-022-00731-5 PMC9970876 36703017

[34] Stefan N, Häring HU, Schulze MB (2013). Metabolically healthy obesity: epidemiology, mechanisms, and clinical implications. Lancet Diabetes Endocrinol.

[35] Tchernof A, Després JP (2013). Pathophysiology of human visceral obesity: an update. Physiol Rev.

[36] Gómez-Ambrosi J, Salvador J, Páramo JA, Orbe J, de Irala J (2002). Involvement of leptin in the association between percentage of body fat and cardiovascular risk factors. Clin Biochem.

[37] Gómez-Ambrosi J, Catalán V, Ramírez B, Rodríguez A, Colina I (2007). Plasma osteopontin levels and expression in adipose tissue are increased in obesity. J Clin Endocrinol Metab.

[38] Catalán V, Gómez-Ambrosi J, Rodríguez A, Ramírez B, Rotellar F (2011). Increased levels of calprotectin in obesity are related to macrophage content: impact on inflammation and effect of weight loss. Mol Med.

[39] Rodríguez A, Gómez-Ambrosi J, Catalán V, Rotellar F, Valentí V (2012). The ghrelin O-acyltransferase-ghrelin system reduces TNF-a-induced apoptosis and autophagy in human visceral adipocytes. Diabetologia.

[40] Lancha A, López-Garrido S, Rodríguez A, Catalán V, Ramírez B (2015). Expression of syntaxin 8 in visceral adipose tissue is increased in obese patients with type 2 diabetes and related to markers of insulin resistance and inflammation. Arch Med Res.

[41] Frühbeck G, Catalán V, Ramírez B, Valentí V, Becerril S (2022). Serum levels of IL-1RA increase with obesity and type 2 diabetes in relation to adipose tissue dysfunction and are reduced after bariatric surgery in parallel to adiposity. J Inflamm Res.

[42] Blüher M (2020). Metabolically healthy obesity. Endocr Rev.

[43] Wildman RP, Muntner P, Reynolds K, McGinn AP, Rajpathak S (2008). The obese without cardiometabolic risk factor clustering and the normal weight with cardiometabolic risk factor clustering: prevalence and correlates of 2 phenotypes among the US population (NHANES 1999–2004). Arch Intern Med.

[44] Vecchie A, Dallegri F, Carbone F, Bonaventura A, Liberale L (2018). Obesity phenotypes and their paradoxical association with cardiovascular diseases. Eur J Intern Med.

[45] Stefan N, Schick F, Haring HU (2017). Causes, characteristics, and consequences of metabolically unhealthy normal weight in humans. Cell Metab.

[46] Velho S, Paccaud F, Waeber G, Vollenweider P, Marques-Vidal P (2010). Metabolically healthy obesity: different prevalences using different criteria. Eur J Clin Nutr.

[47] Primeau V, Coderre L, Karelis AD, Brochu M, Lavoie ME (2011). Characterizing the profile of obese patients who are metabolically healthy. Int J Obes.

[48] Ortega FB, Lee DC, Katzmarzyk PT, Ruiz JR, Sui X (2013). The intriguing metabolically healthy but obese phenotype: cardiovascular prognosis and role of fitness. Eur Heart J.

[49] van Vliet-Ostaptchouk JV, Nuotio ML, Slagter SN, Doiron D, Fischer K (2014). The prevalence of metabolic syndrome and metabolically healthy obesity in Europe: a collaborative analysis of ten large cohort studies. BMC Endocr Disord.

[50] Caleyachetty R, Thomas GN, Toulis KA, Mohammed N, Gokhale KM (2017). Metabolically healthy obese and incident cardiovascular disease events among 3.5 million men and women. J Am Coll Cardiol.

[51] Lavie CJ, Laddu D, Arena R, Ortega FB, Alpert MA (2018). Healthy weight and obesity prevention: JACC health promotion series. J Am Coll Cardiol.

[52] Schulze MB (2019). Metabolic health in normal-weight and obese individuals. Diabetologia.

[53] Smith GI, Mittendorfer B, Klein S (2019). Metabolically healthy obesity: facts and fantasies. J Clin Invest.

[54] Liu J, Zhang Y, Lavie CJ, Moran AE. Trends in metabolic phenotypes according to body mass index among US adults, 1999–2018. Mayo Clin Proc. 2022;97:1664–1679. 10.1016/j.mayocp.2022.02.013 35691704

[55] Marques-Vidal P, Velho S, Waterworth D, Waeber G, von Kanel R (2012). The association between inflammatory biomarkers and metabolically healthy obesity depends of the definition used. Eur J Clin Nutr.

[56] Ortega FB, Cadenas-Sanchez C, Migueles JH, Labayen I, Ruiz JR (2018). Role of physical activity and fitness in the characterization and prognosis of the metabolically healthy obesity phenotype: a systematic review and meta-analysis. Prog Cardiovasc Dis.

[57] Iacobini C, Pugliese G, Blasetti Fantauzzi C, Federici M, Menini S (2019). Metabolically healthy versus metabolically unhealthy obesity. Metabolism.

[58] Loos RJF, Kilpelainen TO (2018). Genes that make you fat, but keep you healthy. J Intern Med.

[59] Camhi SM, Katzmarzyk PT (2014). Differences in body composition between metabolically healthy obese and metabolically abnormal obese adults. Int J Obes (Lond).

[60] Xia L, Dong F, Gong H, Xu G, Wang K (2017). Association between indices of body composition and abnormal metabolic phenotype in normal-weight chinese adults. Int J Environ Res Public Health.

[61] Stefan N, Haring HU, Schulze MB (2018). Metabolically healthy obesity: the low-hanging fruit in obesity treatment?. Lancet Diabetes Endocrinol.

[62] Gómez-Ambrosi J, Catalán V, Rodríguez A, Andrada P, Ramírez B (2014). Increased cardiometabolic risk factors and inflammation in adipose tissue in obese subjects classified as metabolically healthy. Diabetes Care.

[63] Bell JA, Kivimaki M, Hamer M (2014). Metabolically healthy obesity and risk of incident type 2 diabetes: a meta-analysis of prospective cohort studies. Obes Rev.

[64] Chang Y, Kim BK, Yun KE, Cho J, Zhang Y (2014). Metabolically-healthy obesity and coronary artery calcification. J Am Coll Cardiol.

[65] Fan J, Song Y, Chen Y, Hui R, Zhang W (2013). Combined effect of obesity and cardio-metabolic abnormality on the risk of cardiovascular disease: a meta-analysis of prospective cohort studies. Int J Cardiol.

[66] Eckel N, Li Y, Kuxhaus O, Stefan N, Hu FB (2018). Transition from metabolic healthy to unhealthy phenotypes and association with cardiovascular disease risk across BMI categories in 90 257 women (the Nurses’ Health Study): 30 year follow-up from a prospective cohort study. Lancet Diabetes Endocrinol.

[67] Sun M, Fritz J, Haggstrom C, Bjorge T, Nagel G (2023). Metabolically (un)healthy obesity and risk of obesity-related cancers: a pooled study. J Natl Cancer Inst.

[68] Chang Y, Ryu S, Suh BS, Yun KE, Kim CW (2012). Impact of BMI on the incidence of metabolic abnormalities in metabolically healthy men. Int J Obes (Lond).

[69] Soriguer F, Gutiérrez-Repiso C, Rubio-Martín E, García-Fuentes E, Almaraz MC (2013). Metabolically healthy but obese, a matter of time? Findings from the prospective pizarra study. J Clin Endocrinol Metab.

[70] Lin L, Zhang J, Jiang L, Du R, Hu C (2020). Transition of metabolic phenotypes and risk of subclinical atherosclerosis according to BMI: a prospective study. Diabetologia.

[71] Appleton SL, Seaborn CJ, Visvanathan R, Hill CL, Gill TK (2013). Diabetes and cardiovascular disease outcomes in the metabolically healthy obese phenotype: a cohort study. Diabetes Care.

[72] Morkedal B, Vatten LJ, Romundstad PR, Laugsand LE, Janszky I (2014). Risk of myocardial infarction and heart failure among metabolically healthy but obese individuals. The HUNT study, Norway. J Am Coll Cardiol.

[73] Hinnouho GM, Czernichow S, Dugravot A, Batty GD, Kivimaki M (2013). Metabolically healthy obesity and risk of mortality: does the definition of metabolic health matter?. Diabetes Care.

[74] Kramer CK, Zinman B, Retnakaran R (2013). Are metabolically healthy overweight and obesity benign conditions?: a systematic review and meta-analysis. Ann Intern Med.

[75] Jensen MD, Ryan DH, Apovian CM, Ard JD, Comuzzie AG (2014). 2013 AHA/ACC/TOS guideline for the management of overweight and obesity in adults: a report of the American College of Cardiology/American Heart Association Task Force on Practice Guidelines and the obesity society. Circulation.

[76] Yumuk V, Frühbeck G, Oppert JM, Woodward E, Toplak H (2014). An EASO position statement on multidisciplinary obesity management in adults. Obes Facts.

[77] Neeland IJ, Poirier P, Despres JP (2018). Cardiovascular and metabolic heterogeneity of obesity: clinical challenges and implications for management. Circulation.

[78] Pérez-Pevida B, Núñez-Cordoba JM, Romero S, Miras AD, Ibañez P (2019). Discriminatory ability of anthropometric measurements of central fat distribution for prediction of post-prandial hyperglycaemia in patients with normal fasting glucose: the DICAMANO Study. J Transl Med.

[79] Vague J (1956). The degree of masculine differentiation of obesities: a factor determining predisposition to diabetes, atherosclerosis, gout, and uric calculous disease. Am J Clin Nutr.

[80] Pischon T, Boeing H, Hoffmann K, Bergmann M, Schulze MB (2008). General and abdominal adiposity and risk of death in Europe. N Engl J Med.

[81] Cerhan JR, Moore SC, Jacobs EJ, Kitahara CM, Rosenberg PS et al. A pooled analysis of waist circumference and mortality in 650,000 adults. Mayo Clin Proc. 2014;89:335–345. 10.1016/j.mayocp.2013.11.011 PMC4104704 24582192

[82] Lassale C, Tzoulaki I, Moons KGM, Sweeting M, Boer J (2018). Separate and combined associations of obesity and metabolic health with coronary heart disease: a pan-european case-cohort analysis. Eur Heart J.

[83] Ross R, Neeland IJ, Yamashita S, Shai I, Seidell J (2020). Waist circumference as a vital sign in clinical practice: a Consensus Statement from the IAS and ICCR Working Group on visceral obesity. Nat Rev Endocrinol.

[84] Ardern CI, Janssen I, Ross R, Katzmarzyk PT (2004). Development of health-related waist circumference thresholds within BMI categories. Obes Res.

[85] Janssen I, Katzmarzyk PT, Ross R (2002). Body mass index, waist circumference, and health risk: evidence in support of current National Institutes of Health guidelines. Arch Intern Med.

[86] Ardern CI, Katzmarzyk PT, Janssen I, Ross R (2003). Discrimination of health risk by combined body mass index and waist circumference. Obes Res.

[87] Yusuf S, Hawken S, Ounpuu S, Bautista L, Franzosi MG (2005). Obesity and the risk of myocardial infarction in 27,000 participants from 52 countries: a case-control study. Lancet.

[88] Nazare JA, Smith J, Borel AL, Aschner P, Barter P (2015). Usefulness of measuring both body mass index and waist circumference for the estimation of visceral adiposity and related cardiometabolic risk profile (from the INSPIRE ME IAA study). Am J Cardiol.

[89] Vazquez G, Duval S, Jacobs DR, Silventoinen K (2007). Comparison of body mass index, waist circumference, and waist/hip ratio in predicting incident diabetes: a meta-analysis. Epidemiol Rev.

[90] Qiao Q, Nyamdorj R (2010). Is the association of type II diabetes with waist circumference or waist-to-hip ratio stronger than that with body mass index?. Eur J Clin Nutr.

[91] de Koning L, Merchant AT, Pogue J, Anand SS (2007). Waist circumference and waist-to-hip ratio as predictors of cardiovascular events: meta-regression analysis of prospective studies. Eur Heart J.

[92] Czernichow S, Kengne AP, Stamatakis E, Hamer M, Batty GD (2011). Body mass index, waist circumference and waist-hip ratio: which is the better discriminator of cardiovascular disease mortality risk?: evidence from an individual-participant meta-analysis of 82 864 participants from nine cohort studies. Obes Rev.

[93] Aune D, Sen A, Norat T, Janszky I, Romundstad P (2016). Body mass index, abdominal fatness and heart failure incidence and mortality: a systematic review and dose-response meta-analysis of prospective studies. Circulation.

[94] Després JP, Lemieux I, Prud’homme D (2001). Treatment of obesity: need to focus on high risk abdominally obese patients. BMJ.

[95] Ashwell M, Cole TJ, Dixon AK (1996). Ratio of waist circumference to height is strong predictor of intra-abdominal fat. BMJ.

[96] Ashwell M, Gibson S (2014). A proposal for a primary screening tool: ‘keep your waist circumference to less than half your height’. BMC Med.

[97] Savva SC, Lamnisos D, Kafatos AG (2013). Predicting cardiometabolic risk: waist-to-height ratio or BMI. A meta-analysis. Diabetes Metab Syndr Obes.

[98] Gruson E, Montaye M, Kee F, Wagner A, Bingham A (2010). Anthropometric assessment of abdominal obesity and coronary heart disease risk in men: the PRIME study. Heart.

[99] Browning LM, Hsieh SD, Ashwell M (2010). A systematic review of waist-to-height ratio as a screening tool for the prediction of cardiovascular disease and diabetes: 0.5 could be a suitable global boundary value. Nutr Res Rev.

[100] Ashwell M, Gunn P, Gibson S (2012). Waist-to-height ratio is a better screening tool than waist circumference and BMI for adult cardiometabolic risk factors: systematic review and meta-analysis. Obes Rev.

[101] Ashwell M, Gibson S (2016). Waist-to-height ratio as an indicator of ‘early health risk’: simpler and more predictive than using a ‘matrix’ based on BMI and waist circumference. BMJ Open.

[102] Hwaung P, Heo M, Kennedy S, Hong S, Thomas DM (2020). Optimum waist circumference-height indices for evaluating adult adiposity: an analytic review. Obes Rev.

[103] Müller MJ, Braun W, Enderle J, Bosy-Westphal A, Beyond BMI (2016). Conceptual issues related to overweight and obese patients. Obes Facts.

[104] Gonzalez MC, Correia M, Heymsfield SB (2017). A requiem for BMI in the clinical setting. Curr Opin Clin Nutr Metab Care.

[105] Holmes CJ, Racette SB (2021). The utility of body composition assessment in nutrition and clinical practice: an overview of current methodology. Nutrients.

[106] Kahn SE, Hull RL, Utzschneider KM (2006). Mechanisms linking obesity to insulin resistance and type 2 diabetes. Nature.

[107] Favaretto F, Bettini S, Busetto L, Milan G, Vettor R (2022). Adipogenic progenitors in different organs: pathophysiological implications. Rev Endocr Metab Disord.

[108] Klein S, Gastaldelli A, Yki-Järvinen H, Scherer PE (2022). Why does obesity cause diabetes?. Cell Metab.

[109] Sakers A, De Siqueira MK, Seale P, Villanueva CJ (2022). Adipose-tissue plasticity in health and disease. Cell.

[110] Rattarasarn C, Leelawattana R, Soonthornpun S, Setasuban W, Thamprasit A (2003). Relationships of body fat distribution, insulin sensitivity and cardiovascular risk factors in lean, healthy non-diabetic thai men and women. Diabetes Res Clin Pract.

[111] Dervaux N, Wubuli M, Megnien JL, Chironi G, Simon A (2008). Comparative associations of adiposity measures with cardiometabolic risk burden in asymptomatic subjects. Atherosclerosis.

[112] Gómez-Ambrosi J, Silva C, Galofré JC, Escalada J, Santos S (2011). Body adiposity and type 2 diabetes: increased risk with a high body fat percentage even having a normal BMI. Obesity.

[113] Gómez-Ambrosi J, Catalán V, Rodríguez A, Salvador J, Frühbeck G (2015). Does body adiposity better predict obesity-associated cardiometabolic risk than body mass index?. J Am Coll Cardiol.

[114] Gómez-Ambrosi J, Moncada R, Valentí V, Silva C, Ramírez B (2015). Cardiometabolic profile related to body adiposity identifies patients eligible for bariatric surgery more accurately than BMI. Obes Surg.

[115] Gómez-Ambrosi J, Andrada P, Valentí V, Rotellar F, Silva C (2017). Dissociation of body mass index, excess weight loss and body fat percentage trajectories after 3 years of gastric bypass: relationship with metabolic outcomes. Int J Obes (Lond).

[116] Segal KR, Dunaif A, Gutin B, Albu J, Nyman A (1987). Body composition, not body weight, is related to cardiovascular disease risk factors and sex hormone levels in men. J Clin Invest.

[117] De Lorenzo A, Del Gobbo V, Premrov MG, Bigioni M, Galvano F (2007). Normal-weight obese syndrome: early inflammation?. Am J Clin Nutr.

[118] Deurenberg P, Andreoli A, Borg P, Kukkonen-Harjula K, de Lorenzo A (2001). The validity of predicted body fat percentage from body mass index and from impedance in samples of five european populations. Eur J Clin Nutr.

[119] De Lorenzo A, Deurenberg P, Pietrantuono M, Di Daniele N, Cervelli V (2003). How fat is obese?. Acta Diabetol.

[120] Romero-Corral A, Somers VK, Sierra-Johnson J, Jensen MD, Thomas RJ (2007). Diagnostic performance of body mass index to detect obesity in patients with coronary artery disease. Eur Heart J.

[121] Bosy-Westphal A, Geisler C, Onur S, Korth O, Selberg O (2006). Value of body fat mass vs anthropometric obesity indices in the assessment of metabolic risk factors. Int J Obes.

[122] Wellens RI, Roche AF, Khamis HJ, Jackson AS, Pollock ML (1996). Relationships between the body Mass Index and body composition. Obes Res.

[123] Williams DP, Going SB, Lohman TG, Harsha DW, Srinivasan SR (1992). Body fatness and risk for elevated blood pressure, total cholesterol, and serum lipoprotein ratios in children and adolescents. Am J Public Health.

[124] Taylor RW, Falorni A, Jones IE, Goulding A (2003). Identifying adolescents with high percentage body fat: a comparison of BMI cutoffs using age and stage of pubertal development compared with BMI cutoffs using age alone. Eur J Clin Nutr.

[125] McCarthy HD, Cole TJ, Fry T, Jebb SA, Prentice AM (2006). Body fat reference curves for children. Int J Obes (Lond).

[126] Després JP, Lemieux I (2006). Abdominal obesity and metabolic syndrome. Nature.

[127] Piché ME, Poirier P, Lemieux I, Després JP (2018). Overview of epidemiology and contribution of obesity and body fat distribution to cardiovascular disease: an update. Prog Cardiovasc Dis.

[128] Neeland IJ, Ross R, Despres JP, Matsuzawa Y, Yamashita S (2019). Visceral and ectopic fat, atherosclerosis, and cardiometabolic disease: a position statement. Lancet Diabetes Endocrinol.

[129] Cornier MA, Despres JP, Davis N, Grossniklaus DA, Klein S (2011). Assessing adiposity: a scientific statement from the American Heart Association. Circulation.

[130] Piché ME, Tchernof A, Després JP (2020). Obesity phenotypes, diabetes, and cardiovascular diseases. Circ Res.

[131] Wajchenberg BL (2000). Subcutaneous and visceral adipose tissue: their relation to the metabolic syndrome. Endocr Rev.

[132] Fox CS, Massaro JM, Hoffmann U, Pou KM, Maurovich-Horvat P (2007). Abdominal visceral and subcutaneous adipose tissue compartments: association with metabolic risk factors in the Framingham Heart Study. Circulation.

[133] Thomas EL, Parkinson JR, Frost GS, Goldstone AP, Dore CJ (2012). The missing risk: MRI and MRS phenotyping of abdominal adiposity and ectopic fat. Obesity.

[134] Lee DC, Shook RP, Drenowatz C, Blair SN (2016). Physical activity and sarcopenic obesity: definition, assessment, prevalence and mechanism. Future Sci OA.

[135] Donini LM, Busetto L, Bauer JM, Bischoff S, Boirie Y (2020). Critical appraisal of definitions and diagnostic criteria for sarcopenic obesity based on a systematic review. Clin Nutr.

[136] Donini LM, Busetto L, Bischoff SC, Cederholm T, Ballesteros-Pomar MD (2022). Definition and diagnostic criteria for sarcopenic obesity: ESPEN and EASO consensus statement. Clin Nutr.

[137] Gao Q, Mei F, Shang Y, Hu K, Chen F (2021). Global prevalence of sarcopenic obesity in older adults: a systematic review and meta-analysis. Clin Nutr.

[138] von Berens A, Obling SR, Nydahl M, Koochek A, Lissner L (2020). Sarcopenic obesity and associations with mortality in older women and men - a prospective observational study. BMC Geriatr.

[139] Zambon Azevedo V, Ponnaiah M, Bel Lassen P, Ratziu V, Oppert JM (2022). A diagnostic proposal for sarcopenic obesity in adults based on body composition phenotypes. Clin Nutr ESPEN.

[140] Kakinami L, Plummer S, Cohen TR, Santosa S, Murphy J (2022). Body-composition phenotypes and their associations with cardiometabolic risks and health behaviours in a representative general US sample. Prev Med.

[141] Kim HK, Lee MJ, Kim EH, Bae SJ, Choe J (2019). Longitudinal changes of body composition phenotypes and their association with incident type 2 diabetes mellitus during a 5-year follow-up in Koreans. Diabetes Metab J.

[142] Muller MJ, Geisler C, Heymsfield SB, Bosy-Westphal A (2018). Recent advances in understanding body weight homeostasis in humans. F1000Res.

[143] Gibson S, Ashwell M (2020). A simple cut-off for waist-to-height ratio (0.5) can act as an indicator for cardiometabolic risk: recent data from adults in the Health Survey for England. Br J Nutr.

[144] Gómez-Ambrosi J, Silva C, Catalán V, Rodríguez A, Galofré JC (2012). Clinical usefulness of a new equation for estimating body fat. Diabetes Care.

[145] Müller MJ, Lagerpusch M, Enderle J, Schautz B, Heller M (2012). Beyond the body mass index: tracking body composition in the pathogenesis of obesity and the metabolic syndrome. Obes Rev.

[146] Glastonbury CA, Pulit SL, Honecker J, Censin JC, Laber S (2020). Machine learning based histology phenotyping to investigate the epidemiologic and genetic basis of adipocyte morphology and cardiometabolic traits. PLoS Comput Biol.

[147] Majmudar MD, Chandra S, Yakkala K, Kennedy S, Agrawal A (2022). Smartphone camera based assessment of adiposity: a validation study. NPJ Digit Med.

[148] Bennett JP, Liu YE, Quon BK, Kelly NN, Leong LT (2022). Three-dimensional optical body shape and features improve prediction of metabolic disease risk in a diverse sample of adults. Obes (Silver Spring).

[149] World Health Organization. Waist Circumference and Waist-Hip Ratio: Report of a WHO Expert Consultation, Geneva, 8–11 December 2008. Geneva, Switzerland: World Health Organization., 2011. Available at: http://www.who.int/iris/handle/10665/44583. Accessed January 4, 2022.

